# Intrathecal Morphine for Enhanced Recovery After Laparoscopic Colorectal Surgery: A Randomized Clinical Trial

**DOI:** 10.1001/jamasurg.2025.5699

**Published:** 2025-12-23

**Authors:** Liquan Zheng, Yali Lu, Xiaofan Lu, Lizhen You, Chuanchuan Yu, Jielan Lai, Mei Xu, Manxiu Xie, Ying Xiao, Renchun Lai

**Affiliations:** 1 Department of Anesthesiology, State Key Laboratory of Oncology in South China, Guangdong Provincial Clinical Research Center for Cancer, Sun Yat-sen University Cancer Center, Guangzhou, China; 2 Department of Medical Statistics, School of Public Health, Sun Yat-sen University, Guangzhou, China; 3 Department of Anesthesiology, The First Affiliated Hospital, Sun Yat-sen University, Guangzhou, China

## Abstract

**Importance:**

Despite the recovery advantages of minimally invasive surgical techniques, moderate to severe pain after laparoscopic colorectal surgery is a common barrier to improving postoperative recovery quality.

**Objective:**

To evaluate whether intrathecal morphine (ITM) combined with transversus abdominis plane block (TAPB) improves postoperative recovery quality after laparoscopic colorectal surgery.

**Design, Setting, and Participants:**

This prospective, double-blind randomized clinical trial was conducted at Sun Yat-sen University Cancer Center between October 15, 2024, and February 15, 2025. Adults scheduled for elective laparoscopic colorectal surgery were randomized 1:1 to ITM or saline placebo. Data were analyzed from March 1, 2025, to March 31, 2025.

**Interventions:**

Both groups received liposomal bupivacaine for TAPB as part of standard enhanced recovery after surgery (ERAS) protocol. The intervention group received ITM, 3 µg/kg, while the control group received intrathecal normal saline.

**Main Outcomes and Measures:**

The primary outcome was the Quality of Recovery 15 (QoR-15) score at 24 hours postoperatively. Secondary outcomes included postoperative pain scores, cumulative opioid consumption (in morphine milligram equivalents [MME]), time to first flatus, time to first ambulation, incidence of adverse effects, and length of hospital stay.

**Results:**

A total of 252 patients were included in the intention-to-treat analysis (mean [SD] age, 58.4 [11.1] years; 112 female patients [44.4%]). At 24 hours postoperatively, the intervention group had significantly higher mean (SE) QoR-15 scores compared to the control group (114.95 [1.04] vs 102.22 [0.76]; mean difference, 12.21; 95% CI, 9.91-14.51; *P* < .001), indicating better recovery quality. Postoperative mean (SD) morphine consumption was lower in the intervention group compared to the control group (4.4 [6.4] MME vs 10.4 [11.1] MME; mean difference, −6.59; 95% CI, −8.88 to −4.31; *P* < .001). The intervention group also had a reduced incidence of nausea (23.8% vs 37.3%; adjusted risk difference, −15.06%; 95% CI, −26.60% to −3.52%; *P* = .01), but a high incidence of pruritus was observed in the intervention group (19.0% vs 3.2%; adjusted risk difference, 15.08%; 95% CI, 7.26%-22.90%; *P* < .001).

**Conclusions and Relevance:**

Per the results of this randomized clinical trial, in laparoscopic colorectal surgery, ITM combined with TAPB can significantly enhance early postoperative recovery and analgesia, albeit with an increased risk of pruritus. This strategy may be a valuable component of multimodal analgesia regimens following laparoscopic colorectal surgery.

**Trial Registration:**

ClinicalTrials.gov Identifier: NCT06636864

## Key Points

**Question:**

Does intrathecal morphine (ITM) combined with transversus abdominis plane block (TAPB) improve postoperative recovery quality in patients undergoing laparoscopic colorectal surgery?

**Findings:**

In this double-blind randomized clinical trial, a total of 252 patients were included in the intention-to-treat analysis. At 24 hours postoperatively, the intervention group had significantly higher mean (SE) Quality of Recovery 15 scores compared to the control group (114.95 [1.04] vs 102.22 [0.76]).

**Meaning:**

In laparoscopic colorectal surgery, results indicate that ITM combined with TAPB significantly enhanced early postoperative recovery and analgesia, albeit with an increased risk of pruritus; this strategy may be a valuable component of multimodal analgesia regimens following laparoscopic colorectal surgery.

## Teaser

This randomized clinical trial evaluates whether intrathecal morphine combined with transversus abdominis plane block improves postoperative recovery quality after laparoscopic colorectal surgery.

## Introduction

Minimally invasive surgery has become the standard for colorectal procedures. However, nearly half of patients experience moderate to severe postoperative pain within enhanced recovery protocols.^1^ This inadequately controlled pain can impede early mobilization, increase the risk of opioid-related adverse events, and prolong hospital stays.^2^

The transversus abdominis plane block (TAPB) is a widely adopted opioid-sparing analgesic following laparoscopic colorectal surgery,^3,4^ yet it does not effectively address the significant visceral pain component inherent to these procedures.^5^ Intrathecal morphine (ITM), in combination with local anesthetics, offers potent visceral analgesia and reduces opioid requirements, but concerns about adverse effects, such as hypotension and delayed mobilization, have limited its use within Enhanced Recovery After Surgery (ERAS) protocols.^6,7,8,9^ While both TAPB and ITM have individually been shown to improve postoperative outcomes,^10,11^ their combined efficacy within ERAS pathways has not been rigorously evaluated.

In this randomized clinical trial, we hypothesized that at 24 hours postoperatively, the combination of ITM and TAPB would be superior to TAPB alone in enhancing the quality of postoperative recovery, as measured by the Quality of Recovery 15 (QoR-15) questionnaire, in patients undergoing laparoscopic colorectal surgery.

## Methods

### Study Setting

This single-center, prospective, double-blind, placebo-controlled randomized clinical trial was conducted at the Sun Yat-sen University Cancer Center between October 15, 2024, and February 15, 2025. All participants provided written informed consent prior to participation. Ethical approval for the study (B2024-514-01) was granted by the Ethics Committee of Sun Yat-sen University Cancer Center. The trial was prospectively registered on ClinicalTrials.gov on October 15, 2024 (NCT06636864; principal investigator: Dr R. Lai), prior to patient enrollment. The first case was enrolled on October 16, 2024. The study adhered to the Consolidated Standards of Reporting Trials (CONSORT) reporting guidelines and was conducted in compliance with the ethical principles outlined in the Declaration of Helsinki and its subsequent amendment. The trial protocol and statistical analysis plan are available in Supplement 1 and Supplement 2, respectively.

### Participants

Patients were eligible if they (1) were scheduled for laparoscopic colorectal surgery, (2) provided consent for combined postoperative analgesia, (3) had an American Society of Anesthesiologists (ASA) physical status classification of I to III, and (4) were aged 18 years or older. Patients were excluded if they (1) declined surgery due to unforeseen circumstances or personal preference preoperatively, (2) had neurological dysfunction, (3) had contraindications to lumbar puncture, (4) had a history of preoperative opioid use, (5) reported baseline pain (assessed via the numerical rating scale [NRS]) with a score greater than 3, or (6) required conversion to open surgery or secondary operation.

### Randomization and Masking

Potential trial participants were identified by a research team member (M. Xu) through electronic medical record screening. Eligible patients were approached, and written informed consent was obtained before surgery. Patients were randomly allocated in a 1:1 ratio to receive either the intervention or the control using Sun Yat-sen University Cancer Center’s interactive web-based response system (IWRS) with simple randomization. A research assistant (L.Y.) accessed IWRS to obtain the group assignment, who communicated the assignment solely to a designated pharmacist. The pharmacist prepared identical 5-mL intrathecal solutions (normal saline or morphine) in 10-mL syringes for administration. To ensure blinding, neither the research assistant nor the pharmacist was involved in any subsequent procedures and data collection. An independent researcher (L.Z.), who was blinded to group allocation and uninvolved in interventions or analysis, collected all outcome data. The participants, care professionals, outcome assessors, and data analysts were blinded after assignment to interventions.

### Intervention

All patients received standard monitoring (pulse oximetry, noninvasive blood pressure, electrocardiography). In lateral decubitus position with flexed hips and knees, ultrasound guidance (convex array probe, paramedian sagittal oblique placement) identified the target intervertebral space (L3-L4 or L4-L5).^12^ Following lidocaine infiltration, 1%, we advanced a pencil-point spinal needle. After confirming cerebrospinal fluid return, patients received intrathecal morphine, 3 µg/kg, or equivalent-volume normal saline.

Following anesthesia induction, all patients received ultrasound-guided bilateral TAPB using both subcostal and lateral approaches with liposomal bupivacaine. The liposomal bupivacaine (266 mg) was diluted with 40 mL of sodium chloride, 0.9%, to achieve a total volume of 60 mL. Under real-time ultrasound guidance, 15 mL of the diluted solution was injected at right subcostal, left subcostal, right lateral, and left lateral TAP sides. All anesthesiologists had met predefined proficiency: each personally performed more than 10 ultrasound-guided TAPB and more than 50 ultrasound-assisted intrathecal injections for postoperative analgesia before the trial.

All patients received preoperative flurbiprofen, 50 mg, unless contraindicated. Standardized intraoperative management was applied in all cases. General anesthesia was induced with dexmedetomidine, 0.5 µg/kg; ciprofol, 0.4 mg/kg; remifentanil target-controlled infusion, 4 ng/mL; and rocuronium, 0.6 mg/kg. Inhaled desflurane or sevoflurane was adjusted to maintain a bispectral index (BIS system) value of 40 to 60, supplemented with additional muscle relaxants as clinically indicated. Continuous monitoring of BIS and nociception index guided titration of anesthetic depth and opioid dosage. A mean arterial blood pressure was maintained within 20% of baseline. Standard postoperative nausea and vomiting (PONV) prophylaxis comprised dexamethasone, 5 mg, and palonosetron, 0.25 mg, with butorphanol tartrate, 0.1 to 0.2 mg, for pruritus prevention.

### Postoperative Management

All patients received standardized postoperative controlled intravenous analgesia (PCIA) via an intravenous analgesia pump. Standardized PCIA (morphine, 1 mg/mL) was programmed with a background infusion rate of 0 mL per hour and 1-mg bolus dose (6-minute lockout; maximum, 15 mg/hour). If the patient’s NRS score exceeded 4, rescue analgesia was provided via PCIA with 1 mg of morphine. In cases of inadequate pain relief, an additional 50 to 100 mg of tramadol was administered. PONV was managed with palonosetron, 0.25 mg, as needed. Pruritus was systematically assessed using a 4-point scale (with 0 indicating none; 1, mild; 2, moderate, requiring medication; and 3, severe, requiring urgent treatment).^13^ Intravenous butorphanol was available for moderate to severe pruritus.

### Outcomes

The primary outcome of this study was the QoR-15 score at 24 hours postoperatively. The QoR-15 score assesses postoperative recovery across the following 5 dimensions: physical comfort, emotional state, physical independence, pain, and support.^14^ A higher QoR-15 score indicates better recovery quality. Based on prior classifications,^15^ QoR-15 scores were categorized as follows for sensitivity analysis: excellent, 136 to 150; good, 122 to 135; moderate, 90 to 121; and poor, 0 to 89. Secondary outcomes included (1) QoR-15 scores at 48 and 72 hours postoperatively; (2) cumulative opioid consumption (in MME) at 24, 48, and 72 hours postoperatively; (3) total intraoperative opioid dosage; (4) NRS scores during rest or movement in the postanesthesia care unit (PACU) or at 24, 48, and 72 hours postoperatively; (5) time to first flatus; (6) time to first ambulation; (7) supplementary analgesic dosage at 24, 48, and 72 hours postoperatively; (8) adverse events (dizziness or headache, nausea or vomiting, pruritus, hypotension) at 24, 48, and 72 hours postoperatively; and (9) length of hospital stay.

### Data Collection

Data sources comprised both electronic and paper-based medical records, along with patient-completed questionnaires. A trained research assistant, supervised by the principal investigator, was responsible for case report form completion and questionnaire administration.

The primary outcome was measured using the QoR-15 questionnaire at 24 hours postoperatively. Opioid consumption was converted to intravenous morphine equivalents using the following conversion ratios: 100:1 with ITM,^16^ 1:10 with intravenous remifentanil, 1:1 with intravenous sufentanil, and 1:10 with of intravenous tramadol.^17^ Opioid usage data were extracted from electronic medical records and patient-controlled analgesia pump records. The NRS score was used for pain assessment. Secondary outcomes, including postoperative nausea and vomiting, dizziness, time to first ambulation, and time to first flatus, were collected through direct patient interviews and recorded by study personnel.

### Sample Size

The primary outcome measure was the QoR-15 score at 24 hours postoperatively. Based on previous literature and the results of pilot experiments, the minimum clinically important difference of the QoR-15 score was determined to be 6.0.^18^ The SD of the QoR-15 score after major surgery ranged from 10 to 16.^19^ Taking a conservative estimate, an SD of 16 was selected. Assuming α = .05 and β = 0.2 (with a power of 80% to detect this difference), calculations were performed using PASS version 21 software (NCSS). The result indicated that 113 patients were required in each group. Considering a 10% dropout rate, our target was a final sample size of 252.

### Statistical Analysis

This analysis adhered to the intention-to-treat (ITT) and per-protocol principle. Continuous quantitative data were described as mean and SD if normally distributed, with between-group comparisons performed using independent *t* tests. Data not normally distributed were summarized as median and interquartile range and analyzed using the Mann-Whitney *U* test. Categorical variables were expressed as frequencies (percentages), with group comparisons conducted via χ^2^ tests or Fisher exact tests, as appropriate. Ordinal data were similarly described as frequencies (percentages) and compared using the Mann-Whitney *U* test.

For the primary outcome, intergroup differences were assessed using a generalized estimating equation (GEE) model implemented via the R package geepack (R Foundation), with an exchangeable correlation structure. The GEE model incorporated the following 4 time points for repeated QoR-15 measurements: baseline (preoperative), postoperative 24 hours, postoperative 48 hours, and postoperative 72 hours. The model incorporated time, group, their interaction (time × group), and adjustments for age, sex, weight, ASA classification, comorbidities, anesthesia duration, and baseline values. Estimated marginal means at each time point were visualized using line plots, either as absolute values or relative changes from baseline.

Binary outcomes were reported as counts (percentages) and analyzed using generalized linear models to estimate rate differences (RDs) with 95% confidence intervals, adjusting for age, sex, weight, ASA status, comorbidities, and anesthesia duration. For ordinal outcomes, between-group comparisons used the Mann-Whitney *U* test, with median differences and 95% confidence intervals calculated. Single-measurement continuous outcomes were analyzed via linear regression, adjusting for the same covariates.

Longitudinal outcomes lacking baseline measurements were evaluated using GEE models (exchangeable correlation) with time, group, their interaction, and covariates (age, sex, weight, ASA, comorbidities, anesthesia duration). Estimated trends were plotted by group. A 2-sided *P* value less than .05 was considered statistically significant. All analyses and visualizations were performed in R version 4.4.1 (R Foundation).

## Results

### Study population

Between October 15, 2024, and February 15, 2025, a total of 325 patients were assessed for eligibility, and 252 patients were randomly assigned either to the control group (126 patients) or the intervention group (126 patients). Overall mean (SD) patient age was 58.4 [11.1] years, and 112 patients (44.4%) were female. No patients were lost to follow-up. Conversion to laparotomy occurred in 4 patients in the control group (3.2%) and 1 patient in the intervention group (0.8%). Secondary operations were required in 2 patients in the control group (1.6%) and 3 patients in the intervention group (2.4%). A total of 252 participants were included in the final ITT analysis (126 in the intervention group and 126 in the control group), with 242 in the per-protocol analysis (122 in the intervention group and 120 in the control group, shown in eTables 1-8 in Supplement 3). Participant flow is detailed in the Figure. Baseline characteristics are presented in Table 1. Intraoperative data and surgical procedure details are presented in eTable 9 in Supplement 3. No complications related to intrathecal puncture were observed. The first attempt success rate of intrathecal puncture was 85%, with an overall success rate of 100%. The median (IQR) time required for intrathecal puncture was 10 minutes (8-13).

**Figure.**
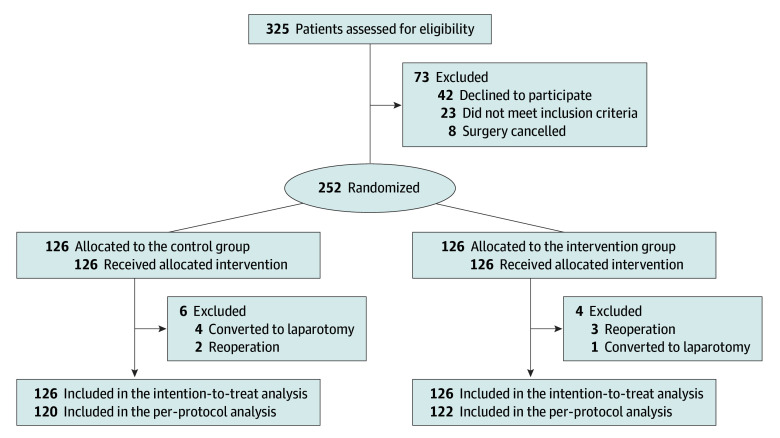
Recruitment, Randomization, and Follow-Up in the Trial

**Table 1.** Baseline and Demographic Characteristics in the Trial

Characteristic	No. (%)	
	Control group (n = 126)	Intervention group (n = 126)
Age, mean (SD), y	60.21 (10.60)	56.57 (11.35)
Sex		
Female	61 (48.4)	51 (40.5)
Male	65 (51.6)	75 (59.5)
BMI, mean (SD)^a^	22.96 (3.27)	23.07 (3.00)
ASA classification		
I	1 (0.8)	5 (4.0)
II	120 (95.2)	121 (96.0)
III	5 (4.0)	0
Hypertension	31 (24.6)	24 (19.0)
Diabetes	5 (4.0)	6 (4.8)
Preoperative chemoradiotherapy	10 (7.9)	12 (9.5)
Preoperative QoR-15 score, mean (SD)	143.18 (4.57)	144.17 (4.81)
Surgery procedure		
Laparoscopic Dixon^b^	24 (19.0)	17 (13.4)
Laparoscopic Dixon + ileostomy^b^	23 (18.3)	37 (29.4)
Laparoscopic Miles^c^	4 (3.2)	2 (1.6)
Laparoscopic transverse colectomy	5 (4.0)	3 (2.4)
Laparoscopic total colectomy	1 (0.8)	0
Laparoscopic sigmoidectomy	29 (23.0)	22 (17.5)
Laparoscopic sigmoidectomy + ileostomy	0	1 (0.8)
Laparoscopic right hemicolectomy	28 (22.22)	26 (20.6)
Laparoscopic right hemicolectomy + ileostomy	1 (0.8)	0
Laparoscopic left hemicolectomy	7 (5.6)	17 (13.5)
Transverse colectomy	1 (0.8)	0
Right hemicolectomy	1 (0.8)	0
Left hemicolectomy	1 (0.8)	0
Dixon^b^	1 (0.8)	1 (0.8)

Abbreviations: ASA, American Society of Anesthesiologists; BMI, body mass index; QoR-15, Quality of Recovery 15.

^a^ Calculated as weight in kilograms divided by height in meters squared.

^b^ Dixon procedure is a sphincter-preserving resection for mid to upper rectal cancers.

^c^ Miles procedure is a combined abdominoperineal resection for low rectal cancers where sphincter preservation is not feasible.

### Primary Outcome

The primary outcome is presented in Table 2. The intervention group had significantly higher mean (SE) QoR-15 scores compared to the control group (114.95 [1.04] vs 102.22 [0.76]; mean difference, 12.21; 95% CI, 9.91-14.51; *P* < .001), indicating better recovery quality. Of the 15 QoR-15 parameters, 14 items (excluding “Getting support from hospital doctors and nurses”) showed significantly higher scores in the intervention group than in the control group (eTable 10 in Supplement 3). Postoperative QoR-15 grading at 24 hours, postoperatively illustrated in the eFigure in Supplement 3, further confirmed the superior recovery quality in the intervention group.

**Table 2.** Quality of Recovery 15 (QoR-15) Global Score at 24, 48, and 72 Hours Postoperatively

Time point	QoR-15 score, mean (SE)		Mean change from baseline (95% CI)		Difference (95% CI)^a^	*P* value
	Control group (n = 126)	Intervention group (n = 126)	Control group	Intervention group		
24 h	102.22 (0.76)	114.95 (1.04)	−41.87 (−43.14 to −40.61)	−29.67 (−31.59 to −27.74)	12.21 (9.91 to 14.51)	<.001
48 h	118.95 (0.72)	124.04 (0.83)	−25.14 (−26.20 to −24.09)	−20.58 (−21.96 to −19.20)	4.56 (2.83 to 6.30)	<.001
72 h	129.38 (0.83)	132.88 (0.81)	−14.71 (−15.87 to −13.54)	−11.75 (−12.82 to −10.67)	2.96 (1.38 to 4.54)	<.001

^a^ Execute the generalized estimating equations (GEE) model using the geepack package in R.

### Secondary Outcomes

At 48 hours postoperatively, the mean total QoR-15 score was significantly higher in the intervention group than in the control group (124.04 vs 118.95; mean difference, 4.56; 95% CI, 2.83-6.30; *P* < .001). This difference persisted at 72 hours postoperatively (132.88 vs 129.38; mean difference, 2.96; 95% CI, 1.38-4.54; *P* < .001) (Table 2; eTables 11 and 12 in Supplement 3).

The intraoperative mean (SD) morphine equivalent in the intervention group was 80.30 (16.70) mg, which was significantly lower than that of 87.30 (29.70) mg in the control group (*P* = .02). The total morphine consumption at 24, 48, and 72 hours postoperatively, as well as during hospitalization, was significantly lower in the intervention group than that in the control group. Morphine equivalents are presented in Table 3.

**Table 3.** Morphine Equivalent Consumption

Consumption	Mean (SD), MME		Difference (95% CI), MME	*P* value
	Control group (n = 126)	Intervention group (n = 126)		
During surgery	87.3 (29.7)	80.3 (16.7)	−6.58 (−11.94 to −1.22)	.02
Postoperatively				
24 h	10.4 (11.1)	4.4 (6.4)	−6.59 (−8.88 to −4.31)	<.001
48 h	13.2 (14.3)	5.8 (7.3)	−8.09 (−10.97 to −5.21)	<.001
72 h	13.9 (15.0)	6.6 (8.0)	−8.02 (−11.06 to −4.97)	<.001
Total	101.2 (34.9)	86.9 (19.5)	−14.61 (−20.96 to −8.26)	<.001

Abbreviation: MME, milligram morphine equivalent.

The intervention group exhibited a significantly lower median (IQR) pain score in the PACU (1.00 [0.00-2.00]) compared to the control group (2.00 [1.00-4.00]) (*P* < .001). The resting pain scores and cough pain scores at 24, 48, and 72 hours postoperatively were significantly lower in the intervention group than those in the control group (eTable 13 in Supplement 3).

Postoperative adverse events are presented in Table 4. At 24 hours postoperatively, compared with the control group, fewer patients in the intervention group experienced nausea (23.8% vs 37.3%; adjusted [adj] RD, −15.06%; 95% CI, −26.60% to −3.52%; *P* = .01) and vomiting (9.5% vs 19.8%; adj RD, −10.35%; 95% CI, −19.17% to −1.52%; *P* = .02). At 48 hours postoperatively, the probability of dizziness was lower in the intervention group (4.8% vs 15.1%; adj RD, −9.82%; 95% CI, −17.31% to −2.34%; *P* = .01). However, compared with the control group, more patients in the intervention group experienced pruritus at 24 hours (19.0% vs 3.2%; adj RD, 15.08%; 95% CI, 7.26%-22.90%; *P* < .001) and 48 hours postoperatively (7.9% vs 1.6%; adj RD, 5.51%; 95% CI, 0.08%-10.94%; *P* = .05).

**Table 4.** Postoperative Adverse Events

Outcome	No. (%)		Unadjusted RD (95% CI)^a^	*P* value	Adjusted RD (95% CI)^a^	*P* value
	Control group (n = 126)	Intervention group (n = 126)				
**Nausea**						
24 h	47 (37.3)	30 (23.8)	−13.49 (−24.79 to −2.19)	.02	−15.06 (−26.60 to −3.52)	.01
48 h	6 (4.8)	5 (4.0)	−0.79 (−5.86 to 4.27)	.76	−0.56 (−5.80 to 4.68)	.84
72 h	3 (2.4)	0	−2.38 (−5.05 to 0.29)	.08	−2.60 (−5.37 to 0.17)	.07
**Vomit**						
24 h	25 (19.8)	12 (9.5)	−10.32 (−19.00 to −1.64)	.02	−10.35 (−19.17 to −1.52)	.02
48 h	2 (1.6)	1 (0.8)	−0.79 (−3.48 to 1.89)	.56	−1.08 (−3.85 to 1.69)	.45
72 h	0	0	NA	NA	NA	NA
**Pruritus**						
24 h	4 (3.2)	24 (19.0)	15.87 (8.33 to 23.41)	<.001	15.08 (7.26 to 22.90)	<.001
48 h	2 (1.6)	10 (7.9)	6.35 (1.13 to 11.57)	.02	5.51 (0.08 to 10.94)	.05
72 h	0	0	NA	NA	NA	NA
**Hypotension**						
24 h	0	4 (3.2)	3.17 (0.10 to 6.25)	.04	3.25 (0.07 to 6.43)	.05
48 h	0	2 (1.6)	1.60 (−0.60 to 3.80)	.16	1.63 (−0.65 to 3.90)	.16
72 h	0	0	NA	NA	NA	NA
**Dizziness**						
24 h	45 (35.7)	38 (30.2)	−5.56 (−17.19 to 6.08)	.35	−5.53 (−17.32 to 6.26)	.35
48 h	19 (15.1)	6 (4.8)	−10.28 (−17.61 to −2.95)	.01	−9.82 (−17.31 to −2.34)	.01
72 h	1 (0.8)	0	−0.79 (−2.35 to 0.76)	.32	−1.02 (−2.63 to 0.60)	.21
**Headache**						
24 h	8 (6.3)	4 (3.2)	−3.17 (−8.44 to 2.09)	.23	−3.83 (−9.29 to 1.63)	.17
48 h	1 (0.8)	0	−0.80 (−2.36 to 0.76)	.32	−0.85 (−2.45 to 0.76)	.30
72 h	0	0	NA	NA	NA	NA

Abbreviations: NA, not applicable; RD, rate difference.

^a^ Analyzed using generalized liner model.

The mean (SD) number of days to first ambulation in the intervention group was significantly lower than in the control group (1.59 [0.53] vs 1.85 [0.47]; *P* < .001). The mean (SD) number of days to the first flatus in the intervention group was significantly lower than in the control group (2.33 [0.55] vs 2.55 [0.60]; *P* = .004). The median (IQR) length of stay was 6.0 days (5.3-7.0) in the intervention group and 6.0 days (6.0-7.0) in the control group (*P* = .11).

## Discussion

This prospective, double-blind randomized clinical trial demonstrated that combining ITM with TAPB significantly enhanced the QoR-15 score in patients undergoing laparoscopic colorectal surgery. The ITM-TAPB regimen was associated with superior postoperative analgesia, reduced opioid requirements, accelerated gastrointestinal recovery, and a favorable adverse effect profile, underscoring its efficacy as a multimodal analgesic strategy within an ERAS framework.

Previous evidence has shown that the addition of ITM with local anesthetics provides robust analgesia compared to systemic opioids,^6,7,8,9,20^ which is consistent with our findings of reduced intraoperative and postoperative morphine requirements and lower pain scores. However, prior studies reported limited improvements in recovery quality,^9,21^ partly due to hypotension and delayed mobilization associated with local anesthetic techniques—factors that conflict with ERAS principles. In contrast, our combined ITM with TAPB protocol showed a lower incidence of postoperative hypotension and fewer opioid-related adverse effects. Most notably, the intervention group achieved clinically meaningful improvements in recovery quality (mean QoR-15 scores: 114.95 vs 102.22; *P* < .001). Additionally, patients exhibited earlier recovery of intestinal function and shorter time to ambulation, indicating enhanced postoperative recovery. These findings confirmed that the combined ITM and TAPB approach aligned with the principles of ERAS in laparoscopic colorectal surgery.

Previous studies have established the efficacy of TAPB in alleviating abdominal wall pain.^10,22^^,^ However, evidence from several studies suggests that TAPB alone may not provide significant analgesic benefits or opioid-sparing effects following laparoscopic abdominal surgery.^23,24^ This discrepancy may arise from 2 key factors. First, TAPB primarily targets somatic pain from surgical incisions but does not adequately address the visceral pain induced. Second, the duration of analgesia with conventional local anesthetics (eg, ropivacaine or bupivacaine) is limited. To overcome these limitations, we combined ITM for visceral pain control with TAPB using liposomal bupivacaine, a formulation designed to prolong analgesic duration. Consistent with prior studies demonstrating the efficacy of liposomal bupivacaine in providing prolonged and effective analgesia,^25,26,27^ our ITM-TAPB regimen reduced opioid consumption and maintained lower pain scores throughout the 72-hour postoperative period, thus confirming its enhanced efficacy.

In the context of ERAS protocols, the primary analgesic objective is to achieve adequate pain control while minimizing adverse effects and accelerating recovery.^28^ Our findings demonstrate that the ITM-TAPB regimen could significantly reduce the incidence of PONV and dizziness compared to TAPB alone. Consistent with prior studies,^6,7,8,9^ pruritus was more frequent in the intervention group. However, its incidence was lower than previously reported, likely due to our conservative dosing strategy^29^ and prophylactic butorphanol tartrate, which may reduce opioid-induced pruritus.^30^

Previous study has demonstrated that ITM doses exceeding 300 µg increase the risk of respiratory depression.^29^ Anatomical study reveals nonuniform distribution of intrathecal medications within cerebrospinal fluid,^31^ enabling equivalent analgesia at lower doses for colorectal surgeries. Based on this pharmacological rationale and preliminary safety data,^32^ we selected a dose of 3 µg/kg ITM for this study, balancing analgesic efficacy with respiratory safety. Current clinical guidelines lack consensus recommendations for ITM in laparoscopic colorectal surgery due to persistent debate about its risk-benefit ratio.^33^ In our study, ultrasound-guided administration achieved 100% procedural success, with no puncture-related complications and reduced overall adverse effects. These findings suggest that the brief additional time required for ITM administration may represent a clinically worthwhile intervention for patients undergoing laparoscopic colorectal procedures.

### Limitations

This study has several limitations. First, as a single-center trial, the generalizability of findings may be constrained, despite an adequate sample size. Second, while we selected a dose of 3 µg/kg of ITM based on preliminary data, the optimal dose remains undefined. Third, the study population was Chinese, with a relatively lower mean body mass index (BMI), so applicability to obese populations is uncertain. Future multicenter trials including more heterogeneous cohorts and a wider range of BMIs are needed to confirm and extend these findings.

## Conclusions

In this randomized clinical trial among patients undergoing laparoscopic colorectal surgery, ITM combined with TAPB significantly enhanced early postoperative recovery and analgesia, albeit with an increased risk of pruritus. This strategy may be a valuable component of multimodal analgesia regimens following laparoscopic colorectal surgery. Future research should focus on determining the ideal ITM dose for laparoscopic colorectal surgery and the combination of ITM with other analgesic modalities.

## Notes

Supplement 1. Trial Protocol

Supplement 2. Statistical Analysis Plan

Supplement 3. eTable 1. Baseline and Demographic Characteristics in the Trial (PP Analysis) eTable 2. Comparison of Perioperative Variables Between Control and Intervention Group (PP Analysis) eTable 3. Primary Outcome: Global, Domain-Specific, and Individual Item Scores on the QoR-15 Scale at 24 h Postoperatively (PP Analysis) eTable 4. Global, Domain-Specific, and Individual Item Scores on the QoR-15 Scale at 48 h Postoperatively (PP Analysis) eTable 5. Global, Domain-Specific, and Individual Item Scores on the QoR-15 Scale at 72 h Postoperatively (PP Analysis) eTable 6. Pain Scores (PP Analysis) eTable 7. Morphine Equivalent Consumption (PP Analysis) eTable 8. Postoperative Adverse Events (PP Analysis) eTable 9. Comparison of Intraoperative Variables Between Control and Intervention Group eTable 10. Global, Domain-Specific, and Individual Item Scores on the QoR-15 Scale at 24 h Postoperatively eTable 11. Global, Domain-Specific, and Individual Item Scores on the QoR-15 Scale at 48 h Postoperatively eTable 12. Global, Domain-Specific, and Individual Item Scores on the QoR-15 Scale at 72 h Postoperatively eTable 13. Pain Scores eFigure. Distribution of Patients According to Categories of Quality of Recovery

Supplement 4. Data Sharing Statement
