# Supplementary material for: Intrathecal Morphine for Enhanced Recovery After Laparoscopic Colorectal Surgery: A Randomized Clinical Trial
Source: JAMA Surg. 2025 Dec 23;161(2):124–31. doi: 10.1001/jamasurg.2025.5699 (PMC12728733; doi:10.1001/jamasurg.2025.5699)
Supplement: Supplement 1. — Trial Protocol [file jamasurg-e255699-s001.pdf]

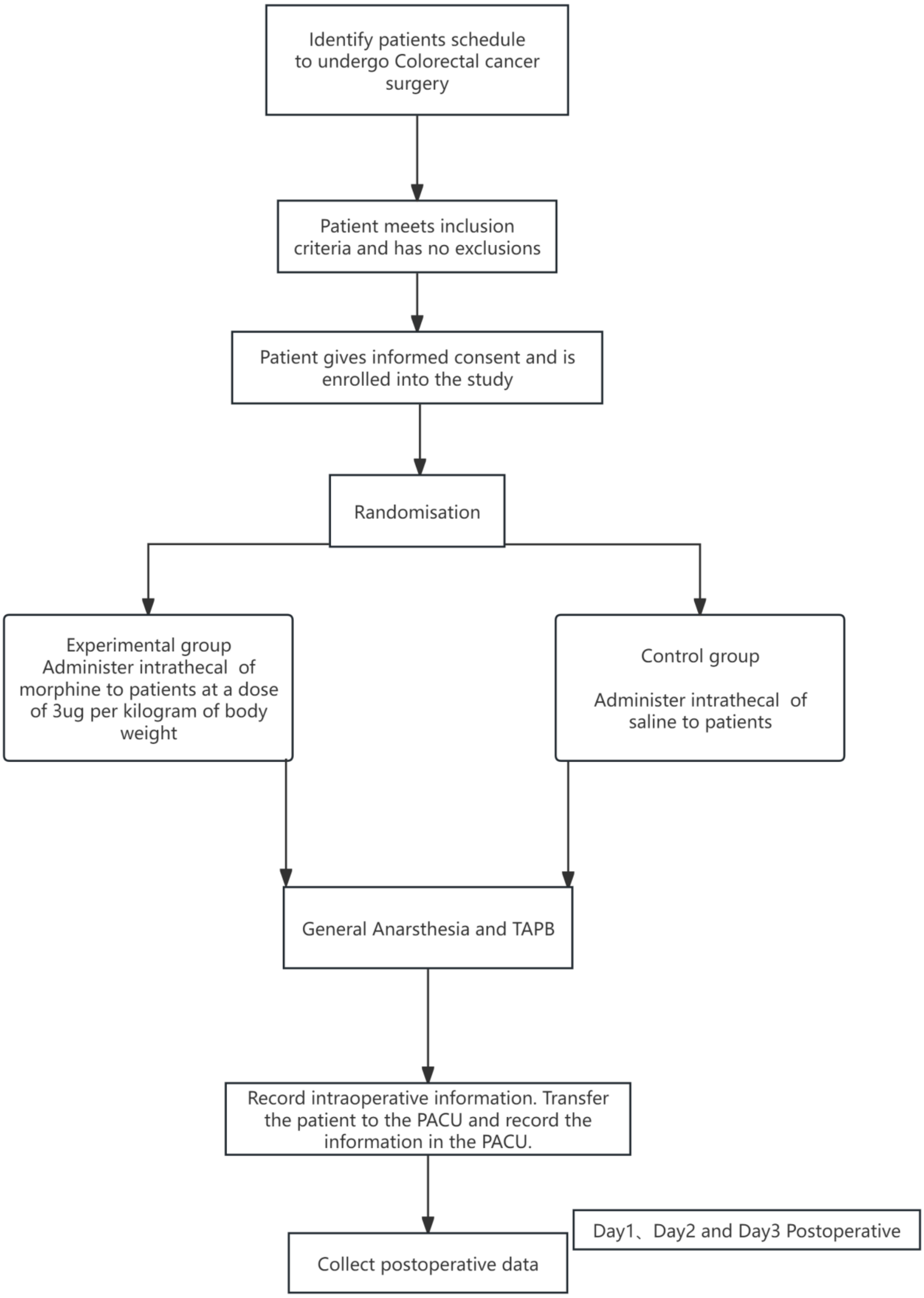

Fig. 1 Study flowchart

Eligibility criteria

The eligibility criteria for patients to be enrolled in this study are as follows:

Inclusion criteria:

a) Planned to undergo laparoscopic colorectal cancer surgery under general anesthesia with endotracheal intubation.

b) Agree to use intrathecal morphine injection and liposomal bupivacaine transversus abdominis plane block after surgery.

c) American Society of Anesthesiologists (ASA) physical status classification of grade I - III.

d) Over 18 years old.

Exclusion criteria:

a) Those who refused the surgery accidentally or subjectively before the operation.

b) b) Patients with neurological dysfunction.

c) Those with contraindications to intraspinal anesthesia.

d) Patients with a history of preoperative opioid use.

e) Patients with abnormal preoperative pain and a pain score (NRS) > 3.

Take informed consent.

In the context of this trial, potential participants will be initially screened and recognized by a designated member of the research team. This team member will meticulously scrutinize the electronic medical records of the patients under consideration to ascertain their eligibility for the trial. Specifically, the determination will be based on whether they fulfill the predefined inclusion criteria and do not fall within the exclusionary parameters.

Should suitable patients be identified, they will be approached either in the anesthesia office on the eve of the operation or on the morning of the surgical procedure. The primary aim of this interaction is to validate their aptitude for participating in the trial. During this encounter, a detailed explanation will be provided to the patients regarding the purpose of the trial, the nature and implications of intrathecal morphine and intrathecal normal saline administration, the details and potential benefits and risks associated with TAPB, as well as the methods of follow-up. A comprehensive and detailed informed consent form will be furnished to each patient, affording them ample time, no less than 5 minutes, to thoroughly understand the contents therein. The subjects will be explicitly informed that their participation in the study is entirely discretionary and that they possess the unrestricted right to withdraw from the study at any stage without any adverse impact on the quality of the treatment they are receiving. Subsequently, a member of the research team will procure the written informed consent form from the subjects, thereby formalizing their entry into the trial protocol.

Interventions

Explanation for the choice of comparators

Intrathecal morphine or salute: The administration of intrathecal morphine or intrathecal normal saline will be executed by proficient anesthesiologists who are blinded to the patient groupings. The ensuing procedures will be implemented for the injection of intrathecal morphine or intrathecal normal saline: Initially, the patient will be positioned in the lateral decubitus position and instructed to flex their hips and knees. Subsequently, a convex ultrasound probe (C5-1S) will be horizontally placed adjacent to the patient's spine. At the anticipated puncture intervertebral space level, such as the L3-L4 or L4-L5 intervertebral space, the target intervertebral space will be precisely identified via the ultrasound image. This space is manifested as a relatively hypoechoic channel within the ultrasound image, with the lamina situated medially and the transverse process laterally. The depth range and entry path for the puncture needle will be determined, and the puncture point will be demarcated on the patient's skin using a

marking pen. The skin will then be cleansed with 2% chlorhexidine gluconate/70% ethanol skin disinfectant, and local anesthesia will be administered at the identified site with 5 ml of 1% lidocaine. Subsequently, a pencil-point spinal needle will be utilized for the puncture. Upon experiencing two sensations of breakthrough and the visualization of cerebrospinal fluid outflow, 3 μg/kg of morphine or normal saline will be injected.

Transversus abdominis plane block: The implementation of TAPB will be carried out as follows: The patient will be positioned in the supine position, and the skin will be cleansed with 2% chlorhexidine gluconate/70% ethanol skin disinfectant. The linear ultrasound probe (L15-4B) will be inserted into a sterile cover and placed transversely at the anterior axillary line between the costal margin and the iliac crest. On the ultrasound image, the skin, subcutaneous tissue, external oblique muscle of the abdomen, internal oblique muscle of the abdomen, and transversus abdominis muscle can be visualized in a sequential manner from the superficial to the deep layer. Under the guidance of ultrasound, the nerve block needle will be inserted and slowly advanced (utilizing the out-of-plane technique) until the needle tip reaches the transversus abdominis plane. Upon proper positioning, liposomal bupivacaine (66.5 mg, 20 ml) will be injected. The identical operation will be replicated on the opposite side. Subsequently, the convex ultrasound probe will be placed beneath the costal margin. On the ultrasound image, the subcutaneous tissue, anterior layer of the rectus sheath, rectus abdominis muscle, posterior layer of the rectus sheath, and transversus abdominis muscle can be observed in a consecutive order from the superficial to the deep layer. Under the guidance of ultrasound, the puncture needle will be inserted deeply into the transversus abdominis plane (employing the in-plane technique) and liposomal bupivacaine (66.5 mg, 20 ml) will be injected. The same procedure will be executed on the opposite side.

Criteria for discontinuing or modifying allocated interventions

The criteria for discontinuing the study are as follows:

a) Those who fail the intrathecal morphine injection or the transversus abdominis plane block.

b) When hypotension persists after the use of vasopressors in patients.

c) When motor paralysis occurs due to intrathecal morphine injection, the study will be stopped.

d) Subjects can withdraw from the study at any time for any reason.

e) In case the individual risk/benefit ratio for a subject becomes unacceptable, the researcher has the right and responsibility to stop the study.

f) If a subject withdraws the informed consent form, they must withdraw from the study.

Strategies to improve adherence to interventions

The study flowchart (Figure 1) will be furnished to the operating room, the surgeons and anesthesiologists responsible for performing the interventions, the Post Anesthesia Care Unit (PACU) department, and the data collectors to ensure adherence to the protocol. "Compliance education" will be implemented for the subjects and their family members to apprise them of the significance of following medical advice and the potential ramifications of non-compliance. Additionally, they will be informed about the knowledge of the disease and the advantages that the treatment can offer. By this means, patients can secure the guarantee of medical monitoring and psychological support, thus enhancing their compliance.

Relevant concomitant care permitted or prohibited during the trial

All patients will undergo standard combined intravenous-inhalation anesthesia with endotracheal intubation. Upon entry into the operating room, the heart rate, electrocardiogram, pulse oxygen saturation, non-invasive blood pressure, and end-tidal carbon dioxide partial pressure (ETCO2) will be routinely monitored. For the induction of general anesthesia, dexmedetomidine (0.5 μg/kg), ciprofol (0.4 mg/kg), remifentanil (administered via target-controlled infusion at 4 ng/ml), and rocuronium bromide (0.6 mg/kg) will be intravenously administered for induction, subsequent to which endotracheal intubation will be performed. Anesthesia maintenance will be accomplished with 1 - 1.3 minimum alveolar concentration (MAC) of desflurane, and remifentanil, cisatracurium, and vasoactive drugs will

be supplemented as dictated by the clinical situation. The intraoperative dosage of opioids will be meticulously recorded.

Patients in both groups will be provided with patient-controlled intravenous analgesia. The intravenous analgesic pump will consist of 150 mg of morphine diluted with 0.9% sodium chloride solution to a total volume of 150 ml. The background infusion rate will be set at 0 ml/h, and the single patient-controlled analgesia dose will be 1 ml, with a lockout time of 6 minutes to manage breakthrough pain.

Provisions for post-trial care

If any participant suffers from any complications arising directly from either intervention,he/she will receive standard postoperative management which may include from the surgical team, pain medicine department and allied health care professions.

Participant timeline

The schedule for enrollment, intervention, and evaluation is shown in Figure 2.

	Study Period							
	Enrolment	Allocation	Post-allocation				Close-out	
Timepoint	Day before surgery	Day of surgery	PACU	Post op Day1	Post op Day2	Post op Day3	Length of hospital stay	Length of hospital stay after surgery
ENROLMRNT								
Eligibility screen	×							
Informed consent	×							
Medical history taking	×							
Allocation		×						
INTERVENTIONS								
Intrathecal morphine or salute		×						
Transversus abdominis plane block		×						
ASSESSMENTS								
QoR-15 scores	×			×		×		
NRS			×	× ——	——	—— ×		
Total opioid consumption				× ——	——	—— ×		
Time of the first flatus					×			
Time of the first ambulation					×			
Amount of analgesic drugs supplemented			X	× ——	——	—— ×		
Adverse events				× ——	——	—— ×		
Length of hospital stay							×	×

Fig. 2 Time schedule of enrolment, interventions, assessments and visits for participants

Recruitment

Members of the anesthesia and research teams from the clinical research center will participate in the recruitment process.

Assignment of interventions: allocation

Patients were randomly allocated in a 1:1 ratio to receive either the intervention group or the control group using Sun Yat-sen University Cancer Center's (SYSUCC) interactive web-based response system (IWRS) with simple randomization. Until the completion of the study, the investigators of this trial will have no access to the randomization system. The randomization process will be carried out by research assistants who are not engaged in the implementation of this trial.

The patient study numbers along with group assignments will be printed on separate pages and concealed within sequentially numbered opaque sealed envelopes. Subsequently, these envelopes will be provided to the operating room pharmacists who will be responsible for preparing the appropriate intrathecal solutions. Based on the sequentially numbered random list, the pharmacists will prepare

either normal saline or morphine syringes. Ultimately, a 10-milliliter syringe (with a total volume of 5 milliliters of clear solution) will be delivered to the anesthesia nurse. All the syringes, regardless of whether they contain normal saline or morphine solution, will have an identical appearance. The anesthesiologist in charge of the intrathecal injection will not take part in the subsequent anesthesia procedures.

Data collection and management

Plans for assessment and collection of outcomes

Data collection will be implemented at three specific time points, namely, prior to surgery, during the surgical procedure, and after surgery. The data will be derived from a combination of information available in both electronic and paper patient medical records, as well as directly collected from patients by means of having them fill out questionnaires. A designated member of the research team will be assigned to take on the principal responsibilities for data collection. This member will remain blinded to the specific groupings of patients, will refrain from participating in the data analysis process, and will not be involved in conducting research interventions. Meanwhile, this data collector will receive training from the principal investigator regarding the procedures for filling out the Case Report Form (CRF) and various questionnaires (such as the QoR-15, NRS, etc.).

The primary outcome will be assessed by having patients complete the QoR-15 questionnaire [12] 24 hours after surgery. The QoR-15 is a 15-parameter questionnaire with scores spanning from 0 to 150. It primarily centers on the recovery status of patients in multiple dimensions, including physical function, psychological state, and pain level following surgery, thereby facilitating medical staff to comprehensively understand the impact of surgery on patients' quality of life from the patients' perspective. The QoR-15 has been recommended as the optimal tool for evaluating the overall recovery of postoperative patients. This scale can effectively differentiate between patients with and without postoperative complications and also exhibits a good correlation with the Global Visual Analogue Recovery Scale. Moreover, it demonstrates good internal consistency, split-half reliability, and test-retest reliability.

The principal secondary outcomes of this trial are as follows: QoR-15 scores at 48 and 72 hours after surgery, morphine consumption equivalents at 24, 48, and 72 hours after surgery, and NRS scores at 24, 48, and 72 hours after surgery. The calculation method for morphine equivalents is detailed as follows: the conversion factor between the milligrams of intravenously injected morphine and the milligrams of intrathecal morphine is 1:100, and the conversion factor with the micrograms of intravenously injected remifentanil is 1:10, and with the micrograms of intravenously injected sufentanil is 1:1. The usage amounts of opioids will be retrieved through electronic medical records and the patient-controlled analgesia pump system. The pain score is measured using the NRS scale, which is characterized by being easy to understand, enabling quantitative assessment, having broad applicability, good internal consistency, higher compliance , and better response intensity.

Data management

Data will initially be collected manually and subsequently transcribed into Microsoft Excel. The data gathered from the clinical research center will be safely stored within the Anesthesiology Department of the research center hospital, on computers that are protected by passwords and can only be accessed by the researchers who are specifically designated for data entry, processing, and analysis. The data will be directly collected from the original documents and recorded into de-identified and coded paper CRF, and then inputted into the electronic CRF.

Confidentiality

All research data will be stored using the research identification numbers assigned to each patient. A page of identifiable patient data that records the assigned patient identification codes will also be separately stored in a locked filing cabinet/office (accessible only to authorized personnel) in order to record postoperative follow-up, supplement missing data points, and allow potential supervisory visits by the national coordinating researcher. This data page will be provided only to the members of the research

team responsible for data entry and the principal investigator. No identifying details of the patients will be reported in any future publications.
