# Supplementary material for: Intrathecal Morphine for Enhanced Recovery After Laparoscopic Colorectal Surgery: A Randomized Clinical Trial
Source: JAMA Surg. 2025 Dec 23;161(2):124–31. doi: 10.1001/jamasurg.2025.5699 (PMC12728733; doi:10.1001/jamasurg.2025.5699)
Supplement: Supplement 2. — Statistical Analysis Plan [file jamasurg-e255699-s002.pdf]

Statistical Analysis Plan

TRIAL FULL TITLE	**Effect of Intrathecal Morphine Combined with Transversus Abdominis Plane Block on Quality of Recovery After Laparoscopic Colorectal Surgery: A Randomized Clinical Trial**
SAP VERSION	1.0
SAP VERSION DATE	2024.09.15
TRIAL STATISTICIAN	Chuanchuan Yu
TRIAL CHIEF INVESTIGATOR	Renchun Lai
SAP AUTHOR	Chuanchuan Yu<br>Department of Medical Statistics, School of Public Health, Sun Yat-sen University, Guangzhou, Guangdong

1 Table of Contents

1 Table of Contents 2
2 Abbreviations and Definitions 3
3 Introduction 3
3.1 Purpose of the analyses 4
4 Study Objectives and Endpoints 4
4.1 Study Objectives 4
4.2 Endpoints 4
5 Study Methods 5
5.1 General Study Design and Plan 5
5.2 Inclusion-Exclusion Criteria and General Study Population 7
5.3 Randomization and Blinding 7
5.4 Study Variables 8
6 Sample Size 11
7 General Considerations 12
7.1 Timing of Analyses 12
7.2 Analysis Populations 12
7.3 Covariates and Subgroups 12
7.4 Missing Data 12
7.5 Interim Analyses and Data Monitoring 12
7.6 Multi-center Studies 13
8 Summary of Study Data 13
8.1 Protocol Deviations 13
8.2 Demographic and Baseline Variables 14
9 Efficacy Analyses 14
9.1 Primary Efficacy Analysis 15
9.2 Secondary Efficacy Analyses 15

9.3 Exploratory Efficacy Analyses 15
10 Safety Analyses 15
11 Figures 16
12 Reporting Conventions 16
13 Technical Details 17

2 Abbreviations and Definitions

ITM	Intrathecal Morphine
TAPB	Transversus Abdominis Plane Block
ERAS	Enhanced Recovery After Surgery
QoR-15	Quality of Recovery-15
MME	Morphine Milligram Equivalents
ITT	Intention-to-treat
CONSORT	Consolidated Standards of Reporting Trials
ASA	American Society of Anesthesiologists
NRS	Numerical Rating Scale
IWRS	Interactive Web-based Response System
TCI	Target-controlled Infusion
BIS	Bispectral Index
NOX	Nociception Index
PONV	Postoperative Nausea and Vomiting
PCIA	Postoperative Controlled Intravenous Analgesia
PACU	Post-anesthesia Care Unit
CRF	Case Report Form
MICD	Minimum Clinically Important Difference
SD	Standard Deviation (SD)
GEE	Generalized Estimating Equation (GEE)
RDs	Rate Differences (RDs)

3 Introduction

This document details the statistical analysis plan for Effect of Intrathecal Morphine Combined with Transversus Abdominis Plane Block on Quality of Recovery After Laparoscopic Colorectal Surgery: A Randomized Clinical Trial. The clinical trial is registered at ClinicalTrials.gov NCT06636864). One should also refer to the trial

protocol paper.

4 Study Objectives and Endpoints

4.1 Study Objectives

The aim of the study is to evaluate whether intrathecal morphine (ITM) combined with transversus abdominis plane block (TAPB) improves postoperative recovery quality after laparoscopic colorectal surgery.

4.2 Endpoints

Primary endpoint:

The primary endpoint is the QoR-15 score at 24 h postoperatively.

Definition : QoR - 15 is a scale used to assess the quality of postoperative recovery. It was developed by McDowell and his team in Sweden in 2005 and is a simplified version of the QoR scale. QoR - 15 consists of 15 items and 5 domains, namely physical state, cognitive state, emotional state, social state, and pain state. It assesses the quality of postoperative recovery by measuring the patient's self - perceived psychological and social function states. Each item is scored on a 10 - point scale, with a total score of 150 points. The higher the score, the better the recovery quality.

Secondary endpoints:

1) QoR-15 scores at 48 and 72 h postoperatively,
2) cumulative opioid consumption (MME) at 24, 48, and 72 h postoperatively,
3) total intraoperative opioid dosage,
4) NRS scores during rest or movement in the post-anesthesia care unit (PACU) or at 24, 48, and 72 h postoperatively,
5) time to first flatus,
6) time to first ambulation,
7) supplementary analgesic dosage at 24, 48, and 72 h postoperatively,
8) adverse events (dizziness/headache, nausea/vomiting, pruritus, hypotension) at 24, 48, and 72 h postoperatively and
9) length of hospital stay.

5 Study Methods

5.1 General Study Design and Plan

This single-center, prospective, randomized, double-blind, placebo-controlled trial was conducted at the Sun Yat-sen University Cancer Center.All participants provided written informed consent prior to participation. Ethical approval for the study (B2024-514-01) was granted by the Ethics Committee of Sun Yat-sen University Cancer Center. The trial was prospectively registered on ClinicalTrials.gov on October 15, 2024(NCT06636864, https://clinicaltrials.gov/study/NCT06636864, principal investigator: Renchun Lai) prior to patient enrollment. The first case was enrolled on October 16, 2024. The study adhered to the Consolidated Standards of Reporting Trials (CONSORT) guidelines and was conducted in compliance with the ethical principles outlined in the Declaration of Helsinki and its subsequent amendment.

	Study Period							
	Enrolment	Allocation	Post-allocation				Close-out	
Timepoint	Day before surgery	Day of surgery	PACU	Post op Day1	Post op Day2	Post op Day3	Length of hospital stay	Length of hospital stay after surgery
ENROLMRNT								
Eligibility screen	×							
Informed consent	×							
Medical history taking	×							
Allocation		×						
INTERVENTIONS								
Intrathecal morphine or salute		×						
Transversus abdominis plane block		×						
ASSESSMENTS								
QoR-15 scores	×			×		×		
NRS			×	×		×		
Total opioid consumption				×		×		
Time of the first flatus					×			
Time of the first ambulation					×			
Amount of analgesic drugs supplemented			X	×		×		
Adverse events				×		×		
Length of hospital stay							×	×

Fig. 2 Time schedule of enrolment, interventions, assessments and visits for participants

5.2 Inclusion-Exclusion Criteria and General Study Population

Inclusion criteria:

Patients will be included if they had:(1) were scheduled for laparoscopic colorectal surgery, (2) provided consent for combined postoperative analgesia, (3) had an American Society of Anesthesiologists (ASA) physical status classification of I to III, and (4) were aged ≥18 years.

Exclusion criteria:

Patients will be excluded if they had:(1) declined surgery due to unforeseen circumstances or personal preference preoperatively, (2) had neurological dysfunction, (3) had contraindications to lumbar puncture, (4) had a history of preoperative opioid use, (5) reported baseline pain (assessed via the numerical rating scale [NRS]) with a score >3, or (6) required conversion to open surgery or secondary operation.

5.3 Randomization and Blinding

Potential trial participants were identified by a research team member through electronic medical record screening. Eligible patients were approached, and written informed consent was obtained either before surgery. Patients were randomly allocated in a 1:1 ratio to receive either the intervention group or the control group using Sun Yat-sen University Cancer Center’s (SYSUCC) interactive web-based response system (IWRS). Allocation concealment was ensured by restricting researcher access to the randomization system until study completion. An independent research assistant conducted randomization using sequentially numbered, opaque envelopes containing group assignments. Based on group assignments, the pharmacist prepared identical 5 mL intrathecal solutions (normal saline or morphine) in 10 mL syringes for administration. To ensure blinding, the administering anesthesiologist was excluded from subsequent procedures and data collection. An independent researcher, blinded to group allocation and uninvolved in interventions or analysis, collected all outcome data.

5.4 Study Variables

QoR-15[1]:QoR - 15 is a scale that comprehensively judges the quality of patients' postoperative recovery by evaluating five aspects of patients: pain, physical comfort, physical independence, psychological support, and emotional state. It has good validity, reliability, and clinical practicality.

NRS:NRS is a concise pain assessment scale that allows patients to quantify their pain intensity using a 0–10 numerical scale, commonly used for rapid measurement of pain severity in clinical settings.

MME:MME is a standard unit used to convert the doses of different opioid analgesics into equivalent morphine doses, facilitating clinical drug dosage adjustment and management.

6 Sample Size

The primary outcome measure was the QoR-15 score at 24 h postoperatively. Based on previous literature and the results of pilot experiments, the minimum clinically important difference (MICD) of the QoR-15 score was determined to be 6.0. The standard deviation (SD) of the QoR-15 score after major surgery ranged from 10 to 16. Taking a conservative estimate, we selected an SD of 16. Assuming $\alpha = 0.05$ and $\beta = 0.2$ (with a power of 80% to detect this difference), calculations were performed using PASS 21 software. The result indicated that 113 patients were required in each group. Considering a 10% dropout rate, our target was a final sample size of 252.

7 General Considerations

Statistical analysis will be conducted in accordance with the plan outlined in the SAP. Statistical analysis will abide by these general statistical principles below.

7.1 Timing of Analyses

After all data have been collected and organized, statistics will be conducted.

7.2 Analysis Populations

Analysis will be performed ‘Intention-to-treat’. All randomized subjects will be included in the final analysis.

7.3 Covariates and Subgroups

Covariates：Age,Gender,Weight,ASA,Complication,Duration of Anesthesia

7.4 Missing Data

The missing values in Full Analysis Set will not be imputed.

7.5 Interim Analyses

Interim analyses are not planned.

7.6 Confidence Intervals and p-values

All estimates of differences between groups would be presented with two-sided 95% confidence intervals unless otherwise stated. Statistical significance will be considered if $p<0.05$ (two-sided).

8 Summary of Study Data

All continuous, normally distributed variables will be summarized using the following descriptive statistics: n (non-missing sample size), mean, standard deviation (SD). All continuous, not-normally distributed variables will be summarized using the following descriptive statistics: n (non-missing sample size), median, interquartile range (IQR). The frequency and percentages (based on the non-missing sample size) of observed levels will be reported for all categorical measures of baseline characteristics. Moreover, for dichotomous outcomes point estimates and 95% CI and absolute differences as difference of percentage points and 95% CI will be calculated. All summary tables will be structured with a column for each treatment in the order (Control, Intervention) and will be annotated with the total population size relevant to that table/treatment.

8.1 Protocol Deviations

Major deviations are defined as follows:

- Wrong treatment in contrast to randomization.
- Violation of inclusion and exclusion criteria.
- Missing informed consent.

8.2 Demographic and Baseline Variables

The following variables are considered to be demographic and baseline:

- Age, sex, height, weight, body mass index.
- History -Hypertension, Diabetes mellitus, Analgesic medications,Espiratory diseases
- ASA
- NRS
- Surgical type
- Duration of surgery, general anesthesia.

9 Efficacy Analyses

This analysis adhered to the intention-to-treat (ITT) and per-protocol principle. Continuous quantitative data were described as mean ± standard deviation if normally distributed, with between-group comparisons performed using independent t-tests. Non-normally distributed data were summarized as median (P25, P75) and analyzed using the Wilcoxon rank-sum test. Categorical variables were expressed as frequencies (percentages), with group comparisons conducted via Chi-square tests or Fisher' s exact test, as appropriate. Ordinal data were similarly described as frequencies (%) and compared using the rank-sum test.

For the primary outcome, intergroup differences were assessed using a generalized estimating equation (GEE) model implemented via the R package geepack, with an exchangeable correlation structure. The model incorporated time, group, their interaction (time × group), and adjustments for age, sex, weight, ASA classification, complications, anesthesia duration, and baseline values. Estimated marginal means at each time point were visualized using line plots, either as absolute values or relative changes from baseline.

Binary outcomes were reported as counts (%) and analyzed using generalized linear models to estimate rate differences (RDs) with 95% CI, adjusting for age, sex, weight, ASA status, complications, and anesthesia duration. For ordinal outcomes, between-group comparisons employed the rank-sum test, with median differences and 95% CI calculated. Single-measurement continuous outcomes were analyzed via

linear regression, adjusting for the same covariates.

Longitudinal outcomes lacking baseline measurements were evaluated using GEE models (exchangeable correlation) with time, group, their interaction, and covariates (age, sex, weight, ASA, complications, anesthesia duration). Estimated trends were plotted by group. All analyses and visualizations were performed in R (v4.4.1).

Results of efficacy analyses will be presented as tables or in text in the final publication(s), including p-values.

9.1 Primary Efficacy Analysis

Analysis of primary endpoint (xx) will be performed in accordance with section 9.

9.2 Secondary Efficacy Analyses

Analyses of secondary endpoints will be performed in accordance with section 9.

9.3 Exploratory Efficacy Analyses

Further analysis of exploratory analyses will be performed in accordance with section 9.

10 Safety Analyses

All adverse events which are directly connected to study procedures will be documented and reported by appropriate statistical methods in accordance with section 9.

11 Reporting Conventions

P-values ≥0.001 will be reported to 3 decimal places; p-values less than 0.001 will be reported as '<0.001'. The mean, standard deviation, and any other statistics other than quantiles, will be reported to one decimal place greater than the original data. Quantiles, such as median, or minimum and maximum will use the same number of decimal places as the original data. Estimated parameters,

not on the same scale as raw observations (e.g. regression coefficients) will be reported to 3 significant figures.

12 Software details

Statistical analyses will be performed using the following computer software. The used version number at the time of writing will be reported.

- The R Project for Statistical Computing (The R Foundation for Statistical Computing, Vienna, Austria).

1. Myles PS, Shulman MA, Reilly J, Kasza J, Romero L. Measurement of quality of recovery after surgery using the 15-item quality of recovery scale: a systematic review and meta-analysis. Br J Anaesth. 2022;128(6):1029-1039. doi: 10.1016/j.bja.2022.03.009.
