# Supplementary material for: Intrathecal Morphine for Enhanced Recovery After Laparoscopic Colorectal Surgery: A Randomized Clinical Trial
Source: JAMA Surg. 2025 Dec 23;161(2):124–31. doi: 10.1001/jamasurg.2025.5699 (PMC12728733; doi:10.1001/jamasurg.2025.5699)
Supplement: Supplement 3. — eTable 1. Baseline and Demographic Characteristics in the Trial (PP Analysis) eTable 2. Comparison of Perioperative Variables Between Control and Intervention Group (PP Analysis) eTable 3. Primary Outcome: Global, Domain-Specific, and Individual Item Scores on the QoR-15 Scale at 24 h Postoperatively (PP Analysis) eTable 4. Global, Domain-Specific, and Individual Item Scores on the QoR-15 Scale at 48 h Postoperatively (PP Analysis) eTable 5. Global, Domain-Specific, and Individual Item Scores on the QoR-15 Scale at 72 h Postoperatively (PP Analysis) eTable 6. Pain Scores (PP Analysis) eTable 7. Morphine Equivalent Consumption (PP Analysis) eTable 8. Postoperative Adverse Events (PP Analysis) eTable 9. Comparison of Intraoperative Variables Between Control and Intervention Group eTable 10. Global, Domain-Specific, and Individual Item Scores on the QoR-15 Scale at 24 h Postoperatively eTable 11. Global, Domain-Specific, and Individual Item Scores on the QoR-15 Scale at 48 h Postoperatively eTable 12. Global, Domain-Specific, and Individual Item Scores on the QoR-15 Scale at 72 h Postoperatively eTable 13. Pain Scores eFigure. Distribution of Patients According to Categories of Quality of Recovery [file jamasurg-e255699-s003.pdf]

Supplemental Online Content

Zheng L, Lu Y, Lu X, et al. Intrathecal morphine for enhanced recovery after laparoscopic colorectal surgery: a randomized clinical trial. *JAMA Surg*. Published online December 23, 2025. doi:10.1001/jamasurg.2025.5699

eTable 1. Baseline and Demographic Characteristics in the Trial (PP Analysis)
eTable 2. Comparison of Perioperative Variables Between Control and Intervention Group (PP Analysis)
eTable 3. Primary Outcome: Global, Domain-Specific, and Individual Item Scores on the QoR-15 Scale at 24 h Postoperatively (PP Analysis)
eTable 4. Global, Domain-Specific, and Individual Item Scores on the QoR-15 Scale at 48 h Postoperatively (PP Analysis)
eTable 5. Global, Domain-Specific, and Individual Item Scores on the QoR-15 Scale at 72 h Postoperatively (PP Analysis)
eTable 6. Pain Scores (PP Analysis)
eTable 7. Morphine Equivalent Consumption (PP Analysis)
eTable 8. Postoperative Adverse Events (PP Analysis)
eTable 9. Comparison of Intraoperative Variables Between Control and Intervention Group
eTable 10. Global, Domain-Specific, and Individual Item Scores on the QoR-15 Scale at 24 h Postoperatively
eTable 11. Global, Domain-Specific, and Individual Item Scores on the QoR-15 Scale at 48 h Postoperatively
eTable 12. Global, Domain-Specific, and Individual Item Scores on the QoR-15 Scale at 72 h Postoperatively
eTable 13. Pain Scores
eFigure. Distribution of Patients According to Categories of Quality of Recovery

This supplemental material has been provided by the authors to give readers additional information about their work.

© 2025 Zheng L et al. *JAMA Surgery*.

eTable 1. Baseline and Demographic Characteristics in the Trial (PP Analysis)

Characteristic	Control group (n = 120)	Intervention group (n = 122)
Age, y	59.99 ± 10.48	56.48 ± 11.51
Sex (male)	60 (50.0)	74 (60.7)
BMI, kg/m^2	22.88 ± 3.25	22.81 ± 2.99
ASA		
I	1 (0.8)	5 (4.0)
II	114 (95.0)	117 (95.9)
III	5 (4.2)	0 (0.0)
Hypertension	31 (25.8)	24 (19.7)
Diabetes	5 (4.2)	6 (4.9)
Preoperative chemoradiotherapy	10(8.3)	13(10.6)
Preoperative QoR-15 score	143.76 ± 0.56	144.37 ± 0.57
Surgery procedure		
Laparoscopic Dixon	22 (18.3)	17 (13.9)
Laparoscopic Dixon + ileostomy	23 (19.2)	37 (30.3)
Laparoscopic Miles	4 (3.3)	2 (1.6)
Laparoscopic transverse colectomy	5 (4.2)	2 (1.6)
Laparoscopic total colectomy	1 (0.8)	0 (0.0)
Laparoscopic sigmoidectomy	29 (24.2)	21 (17.2)
Laparoscopic sigmoidectomy + ileostomy	0 (0.0)	1 (0.8)
Laparoscopic right hemicolectomy	28 (23.4)	25 (20.5)
Laparoscopic right hemicolectomy + ileostomy	1 (0.8)	0 (0.0)
Laparoscopic left hemicolectomy	7 (5.8)	17 (13.9)

Abbreviations: BMI, body mass index; ASA, American society of Anesthesiologists.

Miles procedure is a combined abdominoperineal resection for low rectal cancers where sphincter preservation is not feasible.

© 2025 Zheng L et al. *JAMA Surgery*.

Dixon procedure is a sphincter-preserving resection for mid-to-upper rectal cancers.

Data are presented as the mean ± SD or n (%).

© 2025 Zheng L et al. *JAMA Surgery*.

eTable 2. Comparison of Perioperative Variables Between Control and Intervention Group (PP Analysis)

Variable	Control group (n = 120)	Intervention group (n = 122)	*P* value
Surgical duration (min)	210.00 (180.00, 240.00)	202.50 (167.75, 240.00)	0.35
Duration of anesthesia (min)	240.00 (210.00, 275.00)	240.00 (200.00, 270.00)	0.27
Morphine equivalent consumption (mg)	88.15 ± 30.14	80.24 ± 16.50	0.01
Norepinephrine (ug)	500.00 (272.50, 820.00)	584.50 (310.75, 886.00)	0.18
Number of drainage tubes	2.00 (2.00, 2.00)	2.00 (2.00, 3.00)	0.07

Data are presented as median (P25, P75) or mean ± SD.

© 2025 Zheng L et al. *JAMA Surgery*.

eTable 3. Primary Outcome: Global, Domain-Specific, and Individual Item Scores on the QoR-15 Scale at 24 h Postoperatively (PP Analysis)

	Control group (n = 120)	Intervention group (n = 122)	Control group: mean change from baseline (95% CI)	Intervention group : mean change from baseline (95% CI)	Difference (95% CI)[a]	*P* value
QoR-15 global score (0 to 150）	101.88 (0.76)	115.09 (1.06)	-41.88 (-43.18, -40.59)	-29.28 (-31.23, -27.33)	12.60 (10.27 to 14.94)	< 0.001
QoR-15 item scores						
Able to breathe easily	7.93 (0.10)	8.54 (0.10)	-1.83 (-1.99, -1.67)	-1.23 (-1.39, -1.07)	0.60 (0.38 to 0.83)	< 0.001
Been able to enjoy food	1.65 (0.20)	2.58 (0.19)	-7.39 (-7.72, -7.07)	-6.57 (-6.87, -6.26)	0.83 (0.38 to 1.27)	< 0.001
Feeling rested	6.50 (0.16)	7.77 (0.15)	-2.85 (-3.05, -2.65)	-1.61 (-1.79, -1.42)	1.24 (0.97 to 1.52)	< 0.001
Have had a good sleep	3.46 (0.20)	6.35 (0.19)	-5.57 (-5.91, -5.22)	-2.75 (-3.07, -2.42)	2.82 (2.34 to 3.30)	< 0.001
Able to look after personal toilet and hygiene	6.47 (0.12)	7.30 (0.10)	-3.31 (-3.53, -3.09)	-2.57 (-2.72, -2.43)	0.73 (0.47 to 1.00)	< 0.001

© 2025 Zheng L et al. *JAMA Surgery*.

Able to communicate with family or friends	7.77 (0.09)	8.21 (0.09)	-2.14 (-2.27, -2.02)	-1.72 (-1.83, -1.61)	0.42 (0.26 to 0.59)	< 0.001
Getting support from hospital doctors and nurses	8.34 (0.06)	8.40 (0.07)	-0.93 (-1.07, -0.78)	-0.93 (-1.09, -0.78)	-0.01 (-0.22 to 0.20)	0.93
Able to return to work or usual home activities	3.60 (0.18)	4.92 (0.23)	-6.12 (-6.36, -5.89)	-4.92 (-5.27, -4.56)	1.21 (0.78 to 1.63)	< 0.001
Feeling comfortable and in control	7.04 (0.12)	7.98 (0.12)	-2.48 (-2.66, -2.31)	-1.64 (-1.83, -1.45)	0.84 (0.59 to 1.10)	< 0.001
Having a feeling of general well being	7.78 (0.08)	8.26 (0.09)	-1.58 (-1.69, -1.46)	-1.16 (-1.32, -1.00)	0.41 (0.21 to 0.61)	< 0.001
Moderate pain	7.85 (0.11)	8.93 (0.09)	-2.11 (-2.31, -1.91)	-1.04 (-1.17, -0.91)	1.07 (0.83 to 1.31)	< 0.001
Severe pain	9.60 (0.08)	9.94 (0.04)	-0.35 (-0.54, -0.16)	-0.03 (-0.06, -0.00)	0.32 (0.13 to 0.51)	0.001
Nausea or vomiting	7.33 (0.17)	8.33 (0.14)	-2.82 (-3.16, -2.48)	-1.80 (-2.06, -1.53)	1.02 (0.59 to 1.46)	< 0.001
Feeling worried or anxious	8.07 (0.07)	8.71 (0.10)	-1.22 (-1.35, -1.08)	-0.59 (-0.79, -0.39)	0.63 (0.38 to 0.87)	< 0.001

© 2025 Zheng L et al. *JAMA Surgery*.

Feeling sad or depressed	8.36<br>(0.06)	8.81<br>(0.09)	-1.22<br>(-1.34, -1.10)	-0.76<br>(-0.96, -0.57)	0.45<br>(0.22 to 0.68)	< 0.001

Abbreviations: QoR, quality of recovery; CI, confidence interval.
Data are presented as mean (SE).
[a]Execute the Generalized Estimating Equations (GEE) model using the geepack package in R language.

© 2025 Zheng L et al. *JAMA Surgery*.

eTable 4. Global, Domain-Specific, and Individual Item Scores on the QoR-15 Scale at 48 h Postoperatively (PP Analysis)

	Control group (n = 120)	Intervention group (n = 122)	Control group: mean change from baseline (95% CI)	Intervention group : mean change from baseline (95% CI)	Difference (95% CI)[a]	*P* value
QoR-15 global score (0 to 150）	118.62 (0.72)	124.17 (0.84)	-25.14 (-26.25, -24.04)	-20.20 (-21.56, -18.83)	4.94 (3.19 to 6.70)	< 0.001
QoR-15 item scores						
Able to breathe easily	8.50 (0.10)	8.58 (0.10)	-1.27 (-1.44, -1.10)	-1.19 (-1.36, -1.01)	0.08 (-0.16 to 0.32)	0.53
Been able to enjoy food	4.21 (0.22)	5.98 (0.21)	-4.83 (-5.18, -4.49)	-3.16 (-3.53, -2.80)	1.67 (1.17 to 2.17)	< 0.001
Feeling rested	7.66 (0.15)	8.45 (0.15)	-1.68 (-1.87, -1.49)	-0.93 (-1.13, -0.74)	0.75 (0.48 to 1.02)	< 0.001
Have had a good sleep	5.52 (0.15)	6.88 (0.18)	-3.51 (-3.77, -3.25)	-2.22 (-2.52, -1.92)	1.29 (0.89 to 1.69)	< 0.001
Able to look after personal toilet and hygiene	7.29 (0.09)	7.39 (0.11)	-2.48 (-2.62, -2.34)	-2.49 (-2.65, -2.34)	-0.01 (-0.22 to 0.20)	0.92
Able to communicate with family or friends	8.20 (0.08)	8.42	-1.71	-1.51	0.20	0.01

© 2025 Zheng L et al. *JAMA Surgery*.

		(0.10)	(-1.82, -1.61)	(-1.62, -1.40)	(0.05 to 0.35)	
Getting support from hospital doctors and nurses	8.16 (0.06)	8.06 (0.06)	-1.10 (-1.23, -0.97)	-1.27 (-1.40, -1.14)	-0.17 (-0.35 to 0.01)	0.07
Able to return to work or usual home activities	6.94 (0.17)	7.18 (0.16)	-2.78 (-2.95, -2.61)	-2.66 (-2.81, -2.52)	0.12 (-0.10 to 0.34)	0.29
Feeling comfortable and in control	7.95 (0.10)	8.19 (0.11)	-1.58 (-1.73, -1.42)	-1.43 (-1.61, -1.25)	0.15 (-0.09 to 0.39)	0.22
Having a feeling of general well being	8.42 (0.09)	8.59 (0.10)	-0.94 (-1.09, -0.80)	-0.84 (-1.01, -0.66)	0.11 (-0.12 to 0.34)	0.37
Moderate pain	8.96 (0.10)	9.43 (0.09)	-1.00 (-1.20, -0.80)	-0.54 (-0.65, -0.42)	0.46 (0.24 to 0.69)	< 0.001
Severe pain	9.88 (0.03)	9.93 (0.04)	-0.07 (-0.13, -0.00)	-0.04 (-0.08, 0.00)	0.03 (-0.05 to 0.10)	0.51
Nausea or vomiting	9.10 (0.10)	9.23 (0.10)	-1.05 (-1.22, -0.88)	-0.89 (-1.06, -0.73)	0.16 (-0.08 to 0.39)	0.20
Feeling worried or anxious	8.88 (0.07)	8.96 (0.07)	-0.40 (-0.52, -0.28)	-0.34 (-0.48, -0.21)	0.06 (-0.12 to 0.24)	0.55
Feeling sad or depressed	8.95 (0.07)	9.00 (0.06)	-0.63 (-0.76, -0.51)	-0.57 (-0.67, -0.46)	0.07 (-0.10 to 0.23)	0.42

© 2025 Zheng L et al. *JAMA Surgery*.

Abbreviations: QoR, quality of recovery; CI, confidence interval.

Data are presented as mean (SE).

[a]Execute the Generalized Estimating Equations (GEE) model using the geepack package in R language.

© 2025 Zheng L et al. *JAMA Surgery*.

eTable 5. Global, Domain-Specific, and Individual Item Scores on the QoR-15 Scale at 72 h Postoperatively (PP Analysis)

	Control group (n = 120)	Intervention group (n = 122)	Control group: mean change from baseline (95% CI)	Intervention group : mean change from baseline (95% CI)	Difference (95% CI)[a]	*P* value
QoR-15 global score (0 to 150）	129.18 (0.82)	133.03 (0.82)	-14.58 (-15.74, -13.43)	-11.34 (-12.36, -10.31)	3.25 (1.70 to 4.79)	< 0.001
QoR-15 item scores						
Able to breathe easily	9.12 (0.09)	9.39 (0.10)	-0.65 (-0.82, -0.48)	-0.38 (-0.52, -0.24)	0.27 (0.06, 0.49)	0.01
Been able to enjoy food	6.95 (0.16)	6.92 (0.16)	-2.10 (-2.25, -1.95)	-2.22 (-2.41, -2.03)	-0.12 (-0.37, 0.12)	0.33
Feeling rested	8.02 (0.15)	8.62 (0.16)	-1.33 (-1.51, -1.14)	-0.76 (-0.95, -0.58)	0.56 (0.30, 0.82)	< 0.001
Have had a good sleep	6.01 (0.16)	7.12 (0.18)	-3.02 (-3.29, -2.74)	-1.98 (-2.27, -1.69)	1.04 (0.64, 1.44)	< 0.001
Able to look after personal toilet and hygiene	8.19 (0.14)	8.66 (0.13)	-1.58 (-1.82, -1.35)	-1.21 (-1.44, -0.99)	0.37 (0.05, 0.69)	0.03
Able to communicate with family or friends	8.98 (0.10)	9.28 (0.10)	-0.93 (-1.09, -0.78)	-0.65 (-0.77, -0.53)	0.29 (0.09, 0.48)	0.005

© 2025 Zheng L et al. *JAMA Surgery*.

Getting support from hospital doctors and	8.73	8.75	-0.53	-0.58	-0.05	
nurses	(0.07)	(0.08)	(-0.63, -0.43)	(-0.70, -0.46)	(-0.21, 0.10)	0.50
Able to return to work or usual home	7.36	7.35	-2.37	-2.49	-0.13	
activities	(0.18)	(0.16)	(-2.53, -2.20)	(-2.64, -2.34)	(-0.35, 0.10)	0.27
	8.91	9.19	-0.62	-0.43	0.19	
Feeling comfortable and in control	(0.11)	(0.11)	(-0.76, -0.47)	(-0.58, -0.28)	(-0.02, 0.39)	0.07
	9.04	9.27	-0.32	-0.15	0.17	
Having a feeling of general well being	(0.09)	(0.11)	(-0.43, -0.20)	(-0.33, 0.03)	(-0.05, 0.39)	0.13
	9.69	9.89	-0.27	-0.08	0.19	
Moderate pain	(0.09)	(0.08)	(-0.41, -0.14)	(-0.16, -0.01)	(0.04, 0.34)	0.01
	9.92	9.97	-0.03	-0.00	0.03	
Severe pain	(0.03)	(0.03)	(-0.08, 0.01)	(NaN, NaN)	(-0.01, 0.08)	0.15
	9.72	9.79	-0.43	-0.34	0.10	
Nausea or vomiting	(0.08)	(0.09)	(-0.55, -0.32)	(-0.47, -0.20)	(-0.08, 0.28)	0.29
	9.28	9.42	0.00	0.11	0.11	
Feeling worried or anxious	(0.07)	(0.07)	(-0.10, 0.10)	(0.01, 0.22)	(-0.03, 0.26)	0.13
	9.33	9.44	-0.25	-0.13	0.12	
Feeling sad or depressed	(0.06)	(0.06)	(-0.36, -0.14)	(-0.23, -0.03)	(-0.03, 0.27)	0.12

Abbreviations: QoR, quality of recovery; CI, confidence interval.

Data are presented as mean (SE).

© 2025 Zheng L et al. *JAMA Surgery*.

[a]Execute the Generalized Estimating Equations (GEE) model using the geepack package in R language.

© 2025 Zheng L et al. *JAMA Surgery*.

eTable 6. Pain Scores (PP Analysis)

	Control group (n = 120)	Intervention group (n = 122)	Difference (95% CI)	*P* value
NRS in rest				
POD1	1.54 (0.11)	0.43 (0.11)	-1.11 (-1.30, -0.91)	< 0.001
POD2	1.12 (0.12)	0.44 (0.11)	-0.68 (-0.87, -0.49)	< 0.001
POD3	0.71 (0.10)	0.24 (0.11)	-0.47 (-0.61, -0.33)	< 0.001
NRS in cough				
POD1	2.95 (0.13)	1.66 (0.14)	-1.29 (-1.52, -1.05)	< 0.001
POD2	2.56 (0.13)	1.82 (0.13)	-0.74 (-0.93, -0.55)	< 0.001
POD3	1.97 (0.13)	1.38 (0.13)	-0.59 (-0.75, -0.43)	< 0.001

Abbreviations: NRS, numeric rating scale; CI, confidence interval. POD, postoperative day.

Data are presented as mean (SE).

© 2025 Zheng L et al. *JAMA Surgery*.

eTable 7. Morphine Equivalent Consumption (PP Analysis)

	Control group (n = 120)	Intervention group (n = 122)	Difference (95% CI)	*P* value
During surgery	88.2±30.1	80.2±16.5	-7.50 (-12.95, -2.05)	0.007
Postoperatively				
24 h	10.6±11.4	4.5±6.5	-6.81 (-9.18, -4.44)	< 0.001
48 h	13.5±14.6	5.7±7.3	-8.60 (-11.58, -5.63)	< 0.001
72 h	14.2±15.2	6.5±8.0	-8.54 (-11.69, -5.40)	< 0.001
Total	102.3±35.3	86.7±19.2	-16.04 (-22.48, -9.60)	< 0.001

Abbreviations: CI, confidence interval.

Data are presented as mean ± SD.

© 2025 Zheng L et al. *JAMA Surgery*.

eTable 8. Postoperative Adverse Events (PP Analysis)

Outcome	Control group (n = 120)	Intervention group (n = 122)	Unadj RD[a] (95% CI)	*P* value	Adj RD[a] (95% CI)	*P* value
Nausea						
24 h	44 (36.7%)	29 (23.8%)	-12.90 (-24.40 to -1.40)	0.03	-13.81 (-25.44 to -2.18)	0.02
48 h	5 (4.2%)	5 (4.1%)	-0.07 (-5.10 to 4.97)	0.98	0.30 (-4.92 to 5.51)	0.91
72 h	2 (1.7%)	0 (0.0%)	-1.67 (-3.95 to 0.61)	0.15	-1.96 (-4.35 to 0.43)	0.11
Vomit						
24 h	23 (19.2%)	12 (9.8%)	-9.33 (-18.15 to -0.51)	0.04	-9.14 (-18.11 to -0.17)	0.05
48 h	1 (0.8%)	1 (0.8%)	-0.01 (-2.30 to 2.28)	0.99	-0.29 (-2.64 to 2.06)	0.81
72 h	0 (0.0%)	0 (0.0%)	-	-	-	-
Pruritus						
24 h	4 (3.3%)	24 (19.7%)	16.34 (8.51 to 24.16)	< 0.001	15.59 (7.51 to 23.67)	< 0.001
48 h	2 (1.7%)	10 (8.2%)	6.53 (1.10 to 11.96)	0.02	5.57 (0.10 to 11.05)	0.05
72 h	0 (0.0%)	0 (0.0%)	-	-	-	-
Hypotension						
24 h	0 (0.0%)	4 (3.3%)	3.28 (0.08 to 6.48)	0.05	3.28 (-0.04 to 6.61)	0.05
48 h	0 (0.0%)	2 (1.7%)	1.65 (-0.64 to 3.94)	0.16	1.68 (-0.71 to 4.07)	0.17
72 h	0 (0.0%)	0 (0.0%)	-	-	-	-
Dizziness						
24 h	44 (36.7%)	36 (29.5%)	-7.16 (-19.03 to 4.71)	0.24	-6.48 (-18.48 to 5.51)	0.29
48 h	18 (15.0%)	6 (5.0%)	-10.04 (-17.53 to -2.56)	0.01	-9.20 (-16.85 to -1.55)	0.02
72 h	1 (0.8%)	0 (0.0%)	-0.83 (-2.45 to 0.79)	0.31	-1.11 (-2.80 to 0.58)	0.20
Headache						
24 h	8 (6.7%)	4 (3.3%)	-3.39 (-8.86 to 2.09)	0.23	-3.94 (-9.65 to 1.76)	0.18
48 h	1 (0.8%)	0 (0.0%)	-0.84 (-2.47 to 0.79)	0.31	-0.84 (-2.53 to 0.85)	0.33
72 h	0 (0.0%)	0 (0.0%)	-	-	-	-

Abbreviations: CI, confidence interval; unadj RD, unadjusting rate differences.; Adj RD, adjusting rate differences.

© 2025 Zheng L et al. *JAMA Surgery*.

Data are presented as n (%).

[a]Analyzed useing generalized liner model

© 2025 Zheng L et al. *JAMA Surgery*.

eTable 9. Comparison of Intraoperative Variables Between Control and Intervention Group

	Control group (n = 126)	Intervention group (n = 126)	*P* value
Surgical duration (min)	210.00 (180.00, 250.00)	207.50 (171.25, 240.00)	0.33
Duration of anesthesia (min)	240.00 (210.00, 278.75)	240.00 (200.00, 270.00)	0.27
Morphine equivalent consumption (mg)	87.3 ± 29.7	80.3 ± 16.7	0.02
Norepinephrine (ug)	500.00 (280.00, 815.00)	573.00 (301.75, 880.00)	0.22
Number of drainage tubes	2.00 (2.00, 2.00)	2.00 (2.00, 3.00)	0.11

Data are presented as median (P25, P75) or mean ± SD.

© 2025 Zheng L et al. *JAMA Surgery*.

eTable 10. Global, Domain-Specific, and Individual Item Scores on the QoR-15 Scale at 24 h Postoperatively

	Control group (n = 126)	Intervention group (n = 126)	Control group: mean change from baseline (95% CI)	Intervention group : mean change from baseline (95% CI)	Difference (95% CI)[a]	*P* value
QoR-15 global score (0 to 150）	102.22 (0.76)	114.95 (1.04)	-41.87 (-43.14, -40.61)	-29.67 (-31.59, -27.74)	12.21 (9.91 to 14.51)	< 0.001
QoR-15 item scores						
Able to breathe easily	7.95 (0.10)	8.54 (0.10)	-1.83 (-1.99, -1.67)	-1.25 (-1.40, -1.09)	0.59 (0.36 to 0.81)	< 0.001
Been able to enjoy food	1.64 (0.20)	2.55 (0.19)	-7.42 (-7.74, -7.11)	-6.58 (-6.87, -6.28)	0.84 (0.41 to 1.27)	< 0.001
Feeling rested	6.53 (0.15)	7.78 (0.15)	-2.85 (-3.04, -2.65)	-1.63 (-1.82, -1.45)	1.21 (0.95 to 1.48)	< 0.001
Have had a good sleep	3.51 (0.19)	6.34 (0.19)	-5.58 (-5.91, -5.25)	-2.81 (-3.13, -2.49)	2.77 (2.31 to 3.23)	< 0.001
Able to look after personal toilet and hygiene	6.48 (0.12)	7.29 (0.10)	-3.32 (-3.53, -3.10)	-2.60 (-2.74, -2.45)	0.72 (0.46 to 0.98)	< 0.001
Able to communicate with family or friends	7.79 (0.09)	8.21 (0.09)	-2.12 (-2.24, -1.99)	-1.72 (-1.83, -1.62)	0.40 (0.23 to 0.56)	< 0.001
Getting support from hospital doctors and nurses	8.34 (0.06)	8.40 (0.07)	-0.96 (-1.10, -0.82)	-0.96 (-1.11, -0.81)	-0.00 (-0.21 to 0.21)	1.000
Able to return to work or usual home activities	3.61 (0.18)	4.89 (0.23)	-6.13 (-6.35, -5.90)	-4.95 (-5.30, -4.60)	1.17 (0.76 to 1.59)	< 0.001
Feeling comfortable and in control	7.05 (0.12)	7.94 (0.12)	-2.49 (-2.66, -2.32)	-1.68 (-1.87, -1.50)	0.81 (0.56 to 1.06)	< 0.001

© 2025 Zheng L et al. *JAMA Surgery*.

Having a feeling of general well being	7.81 (0.08)	8.24 (0.09)	-1.59 (-1.71, -1.46)	-1.21 (-1.37, -1.05)	0.38 (0.18 to 0.58)	< 0.001
Moderate pain	7.90 (0.11)	8.90 (0.09)	-2.06 (-2.25, -1.86)	-1.06 (-1.20, -0.93)	0.99 (0.75 to 1.23)	< 0.001
Severe pain	9.61 (0.08)	9.94 (0.04)	-0.33 (-0.51, -0.16)	-0.03 (-0.06, -0.00)	0.30 (0.12 to 0.48)	0.001
Nausea or vomiting	7.37 (0.16)	8.33 (0.14)	-2.78 (-3.11, -2.45)	-1.81 (-2.07, -1.54)	0.97 (0.54 to 1.39)	< 0.001
Feeling worried or anxious	8.08 (0.07)	8.70 (0.10)	-1.23 (-1.36, -1.10)	-0.63 (-0.83, -0.43)	0.60 (0.36 to 0.84)	< 0.001
Feeling sad or depressed	8.37 (0.06)	8.79 (0.09)	-1.21 (-1.33, -1.10)	-0.79 (-0.98, -0.59)	0.43 (0.20 to 0.65)	< 0.001

Abbreviations: QoR, quality of recovery; CI, confidence interval.

Data are presented as the mean (SE).

[a]Execute the Generalized Estimating Equations (GEE) model using the geepack package in R language.

© 2025 Zheng L et al. *JAMA Surgery*.

eTable 11. Global, Domain-Specific, and Individual Item Scores on the QoR-15 Scale at 48 h Postoperatively

	Control group (n = 126)	Intervention group (n = 126)	Control group: mean change from baseline (95% CI)	Intervention group : mean change from baseline (95% CI)	Difference (95% CI)a
QoR-15 global score (0 to 150）	118.95 (0.72)	124.04 (0.83)	-25.14 (-26.20, -24.09)	-20.58 (-21.96, -19.20)	4.56 (2.83 to 6.30)
QoR-15 item scores					
Able to breathe easily	8.50 (0.09)	8.58 (0.10)	-1.29 (-1.45, -1.12)	-1.21 (-1.38, -1.04)	0.08 (-0.16 to 0.31)
Been able to enjoy food	4.25 (0.22)	5.94 (0.21)	-4.81 (-5.15, -4.47)	-3.19 (-3.55, -2.83)	1.62 (1.13 to 2.11)
Feeling rested	7.71 (0.15)	8.41 (0.16)	-1.67 (-1.86, -1.49)	-1.01 (-1.21, -0.80)	0.67 (0.39 to 0.94)
Have had a good sleep	5.59 (0.15)	6.81 (0.18)	-3.50 (-3.76, -3.24)	-2.33 (-2.65, -2.02)	1.17 (0.76 to 1.57)
Able to look after personal toilet and hygiene	7.30 (0.09)	7.38 (0.11)	-2.49 (-2.63, -2.35)	-2.51 (-2.66, -2.36)	-0.02 (-0.22 to 0.19)
Able to communicate with family or friends	8.19 (0.08)	8.42 (0.10)	-1.72 (-1.82, -1.62)	-1.51 (-1.62, -1.40)	0.21 (0.06 to 0.36)
Getting support from hospital doctors and nurses	8.17 (0.06)	8.07 (0.06)	-1.13 (-1.26, -1.01)	-1.29 (-1.42, -1.17)	-0.16 (-0.34 to 0.02)
Able to return to work or usual home activities	6.94 (0.17)	7.17 (0.16)	-2.79 (-2.96, -2.63)	-2.67 (-2.81, -2.54)	0.12 (-0.09 to 0.33)
Feeling comfortable and in control	7.95 (0.10)	8.18 (0.11)	-1.60 (-1.75, -1.44)	-1.44 (-1.62, -1.27)	0.15 (-0.08 to 0.38)
Having a feeling of general well being	8.45 (0.09)	8.58 (0.10)	-0.94 (-1.08, -0.80)	-0.87 (-1.04, -0.69)	0.08 (-0.14 to 0.30)
Moderate pain	9.00 (0.10)	9.41 (0.09)	-0.96 (-1.15, -0.77)	-0.55 (-0.66, -0.44)	0.41 (0.19 to 0.63)
Severe pain	9.88 (0.03)	9.93 (0.04)	-0.06 (-0.12, -0.00)	-0.04 (-0.08, 0.00)	0.02 (-0.05 to 0.10)
Nausea or vomiting	9.11 (0.10)	9.23 (0.10)	-1.04 (-1.21, -0.87)	-0.90 (-1.06, -0.75)	0.13 (-0.10 to 0.37)
Feeling worried or anxious	8.91 (0.07)	8.96 (0.07)	-0.40 (-0.52, -0.29)	-0.37 (-0.50, -0.23)	0.04 (-0.14 to 0.22)

© 2025 Zheng L et al. *JAMA Surgery*.

Feeling sad or depressed	8.95 (0.06)	8.99 (0.06)	-0.63 (-0.75, -0.51)	-0.58 (-0.68, -0.48)	0.05 (-0.11 to 0.21)

Abbreviations: QoR, quality of recovery; CI, confidence interval.

Data are presented as the mean (SE).

[a]Execute the Generalized Estimating Equations (GEE) model using the geepack package in R language.

© 2025 Zheng L et al. *JAMA Surgery*.

eTable 12. Global, Domain-Specific, and Individual Item Scores on the QoR-15 Scale at 72 h Postoperatively

	Control group (n = 126)	Intervention group (n = 126)	Control group: mean change from baseline (95% CI)	Intervention group : mean change from baseline (95% CI)	Difference (95% CI)a	P value
QoR-15 global score (0 to 150）	129.38 (0.83)	132.88 (0.81)	-14.71 (-15.87, -13.54)	-11.75 (-12.82, -10.67)	2.96 (1.38 to 4.54)	< 0.001
QoR-15 item scores						
Able to breathe easily	9.12 (0.09)	9.37 (0.10)	-0.67 (-0.83, -0.51)	-0.41 (-0.55, -0.27)	0.25 (0.04 to 0.47)	0.02
Been able to enjoy food	6.93 (0.16)	6.88 (0.16)	-2.13 (-2.28, -1.98)	-2.25 (-2.43, -2.06)	-0.12 (-0.36 to 0.12)	0.33
Feeling rested	8.04 (0.15)	8.61 (0.15)	-1.34 (-1.52, -1.16)	-0.80 (-0.99, -0.62)	0.54 (0.28 to 0.80)	< 0.001
Have had a good sleep	6.05 (0.16)	7.11 (0.18)	-3.04 (-3.31, -2.77)	-2.04 (-2.33, -1.75)	1.00 (0.60 to 1.40)	< 0.001
Able to look after personal toilet and hygiene	8.20 (0.14)	8.63 (0.13)	-1.60 (-1.82, -1.37)	-1.26 (-1.49, -1.04)	0.33 (0.01 to 0.65)	0.04
Able to communicate with family or friends	8.98 (0.10)	9.28 (0.09)	-0.93 (-1.08, -0.78)	-0.66 (-0.78, -0.54)	0.27 (0.08 to 0.46)	0.01
Getting support from hospital doctors and nurses	8.75 (0.07)	8.74 (0.08)	-0.55 (-0.65, -0.45)	-0.63 (-0.75, -0.50)	-0.08 (-0.24 to 0.08)	0.35
Able to return to work or usual home activities	7.39 (0.18)	7.33 (0.16)	-2.35 (-2.52, -2.18)	-2.51 (-2.66, -2.36)	-0.16 (-0.38 to 0.06)	0.16
Feeling comfortable and in control	8.92 (0.11)	9.16 (0.11)	-0.63 (-0.77, -0.49)	-0.46 (-0.61, -0.31)	0.17 (-0.04 to 0.37)	0.11
Having a feeling of general well being	9.06 (0.09)	9.26 (0.11)	-0.33 (-0.45, -0.22)	-0.19 (-0.37, -0.01)	0.14 (-0.07 to 0.36)	0.19

© 2025 Zheng L et al. *JAMA Surgery*.

Moderate pain	9.69 (0.09)	9.86 (0.08)	-0.26 (-0.39, -0.14)	-0.10 (-0.18, -0.03)	0.16 (0.01 to 0.30)	0.03
Severe pain	9.92 (0.03)	9.97 (0.03)	-0.03 (-0.08, 0.01)	-0.00 (NaN, NaN)	0.03 (-0.01 to 0.08)	0.15
Nausea or vomiting	9.71 (0.08)	9.80 (0.09)	-0.44 (-0.55, -0.32)	-0.33 (-0.47, -0.20)	0.10 (-0.07 to 0.28)	0.25
Feeling worried or anxious	9.29 (0.07)	9.42 (0.07)	-0.02 (-0.11, 0.08)	0.10 (-0.01, 0.20)	0.11 (-0.03 to 0.26)	0.13
Feeling sad or depressed	9.33 (0.06)	9.43 (0.06)	-0.25 (-0.36, -0.15)	-0.14 (-0.24, -0.05)	0.11 (-0.03 to 0.26)	0.13

Abbreviations: QoR, quality of recovery; CI, confidence interval.

Data are presented as the mean (SE).

[a]Execute the Generalized Estimating Equations (GEE) model using the geepack package in R language.

© 2025 Zheng L et al. *JAMA Surgery*.

eTable 13. Pain Scores

	Control group (n = 126)	Intervention group (n = 126)	Difference (95% CI)[a]	*P* value
NRS in rest				
POD1	1.53 (0.11)	0.46 (0.11)	-1.08 (-1.27 to -0.88)	< 0.001
POD2	1.11 (0.12)	0.46 (0.11)	-0.66 (-0.85 to -0.47)	< 0.001
POD3	0.69 (0.10)	0.24 (0.11)	-0.44 (-0.58 to -0.31)	< 0.001
NRS in cough				
POD1	2.94 (0.13)	1.69 (0.14)	-1.26 (-1.49 to -1.02)	< 0.001
POD2	2.54 (0.13)	1.85 (0.13)	-0.68 (-0.87 to -0.50)	< 0.001
POD3	1.95 (0.13)	1.39 (0.13)	-0.56 (-0.72 to -0.40)	< 0.001

Abbreviations: NRS, numeric rating scale; CI, confidence interval. POD, postoperative day.

Data are presented as the mean (SE).

[a]Execute the Generalized Estimating Equations (GEE) model using the geepack package in R language.

© 2025 Zheng L et al. *JAMA Surgery*.

eFigure. Distribution of Patients According to Categories of Quality of Recovery

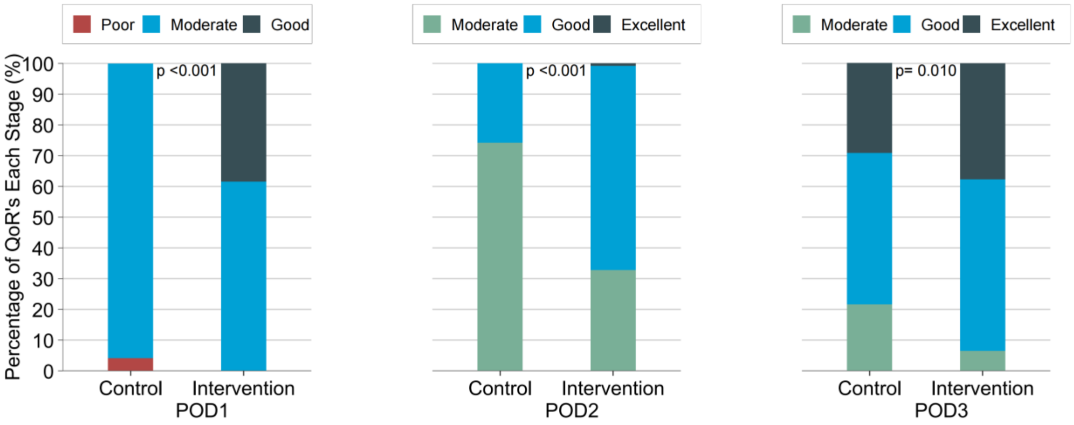

Abbreviations: POD, postoperative day.

© 2025 Zheng L et al. *JAMA Surgery*.
