# Supplementary material for: Intrathecal Morphine for Enhanced Recovery After Laparoscopic Colorectal Surgery: A Randomized Clinical Trial
Source: JAMA Surg. 2025 Dec 23;161(2):124–31. doi: 10.1001/jamasurg.2025.5699 (PMC12728733; doi:10.1001/jamasurg.2025.5699)
Supplement: Supplement 4. — Data Sharing Statement [file jamasurg-e255699-s004.pdf]

Data Sharing Statement

Zheng. Intrathecal Morphine for Enhanced Recovery After Laparoscopic Colorectal Surgery. *JAMA Surg*. Published December 23, 2025. doi:10.1001/jamasurg.2025.5699

Data
Additional Information: https://clinicaltrials.gov/study/NCT06636864
Data available: No
